# A case of Ph^+^ acute lymphoblastic leukemia and EGFR mutant lung adenocarcinoma synchronous overlap: may one TKI drug solve two diseases?

**DOI:** 10.1186/s12920-024-01955-y

**Published:** 2024-07-08

**Authors:** Qi Zhang, Jing-dong Zhou, Hao Ding, Lei Yang, Chao Lu, Ming-qiang Chu, Jun Qian, Ting-juan Zhang

**Affiliations:** 1 Department of Hematology, Affiliated People’s Hospital of Jiangsu University, Zhenjiang, Jiangsu People’s Republic of China; 2 Department of Respiratory Disease, Affiliated People’s Hospital of Jiangsu University, Zhenjiang, Jiangsu People’s Republic of China; 3 Department of Radiology, Affiliated People’s Hospital of Jiangsu University, Zhenjiang, Jiangsu People’s Republic of China; 4 Department of Oncology, Affiliated People’s Hospital of Jiangsu University, 8 Dianli Rd, Zhenjiang, 212002 Jiangsu People’s Republic of China

**Keywords:** Philadelphia positive acute lymphoblastic leukemia, Lung adenocarcinoma, Tyrosine kinase inhibitors, Epidermal growth factor receptor, Case report

## Abstract

**Background:**

Philadelphia chromosome positive (Ph^+^) acute lymphoblastic leukemia (ALL) refers to ALL patients with t(9;22) cytogenetic abnormalities, accounting for about 25% of ALL. Lung adenocarcinoma (LUAD) is the most common pathological type of non-small-cell lung cancer, which has a frequency of approximately 45% cases with mutations in EGFR. Both Ph^+^ ALL and EGFR mutant LUAD are involved in the pathogenesis of the abnormal activation of the tyrosine kinase pathway. Although the second primary hematological malignancy after the treatment of solid tumors is common in clinics, the synchronous multiple primary malignant tumors of hematological malignancy overlap solid tumors are uncommon, even both tumors involved in the pathogenesis of the abnormal activation of the tyrosine kinase pathway are extremely rare.

**Case presentation:**

An 84-year-old man with fatigue and dizziness was diagnosed with Ph^+^ ALL. Meanwhile, a chest CT indicated a space-occupying lesions, characterized by the presence of void, in the right lower lope with the enlargement of mediastinal lymph node and right pleural effusion. After a few weeks, the patient was diagnosed with LUAD with EGFR exon 19 mutation. Both tyrosine kinase inhibitors (TKI) (Flumatinib) and EGFR-TKI (Oxertinib) was used for the patients, and finally have controlled both diseases.

**Conclusion:**

As far as we know, we for the first time reported a case of Ph^+^ ALL and EGFR mutant LUAD synchronous overlap, of which pathogenesis is related to abnormal tyrosine kinase activation. This patient was successfully treated with two different TKIs without serious adverse events.

## Background

Multiple primary malignant tumors (MPM) refer to the occurrence of two or more synchronous or metachronous primary malignant tumors in the same individual. Synchronous MPM is defined as two tumors diagnosed in less than 6 months, whereas metachronous MPM is defined as two tumors diagnosed in more than 6 months. The metachronous MPM occurs more frequently than synchronous MPM in clinics [1]. In the cases that hematological malignances overlap with solid tumors, the most common is metachronous MPM, which presented as the second primary hematological malignancy after the treatment of solid tumors [2]. However, the synchronous MPM of hematological malignancy overlaps solid tumors are uncommon in clinics [3, 4], and both tumors involved in the pathogenesis of the abnormal activation of the tyrosine kinase pathway are extremely rare.

Acute lymphoblastic leukemia (ALL) is a malignant clonal disease arising from the excessive proliferation of B- or T-cell lymphoblasts in the bone marrow [5]. Philadelphia chromosome positive (Ph^+^) ALL refers to ALL patients with t(9;22) cytogenetic abnormalities, a unique subtype of ALL, accounting for about 25% of ALL [6]. Clinical outcome of Ph^+^ ALL remains extremely poor in the absence of tyrosine kinase inhibitors (TKI) [7]. Lung cancer is a type of lung malignant tumor originating from bronchial mucosa, bronchial glands and alveolar epithelium, with a variety of pathological features [8]. Non-small-cell lung cancer (NSCLC) accounts for 80-85% of the lung cancer. The two major histological phenotypes of NSCLC are adenocarcinoma and squamous carcinoma. Lung adenocarcinoma (LUAD) is the most common pathological type of NSCLC. The epidermal growth factor receptor (EGFR) is a transmembrane protein with cytoplasmic kinase activity [9]. When stimulated, transmembrane receptors trigger an intracellular signaling cascade that affects cell proliferation, angiogenesis and apoptosis [9]. Mutations in EGFR are discovered in approximately 45% NSCLC patients, which play as an important pathogenic mechanism of disease occurrence/development, and become as an important therapeutic target [10]. Treatment with EGFR-TKI based therapy in EGFR mutant NSCLC patients achieved satisfactory results [11].

Herein, we reported an elderly case of Ph^+^ ALL synchronous overlap EGFR-mutant LUAD. It is well-known that both Ph^+^ ALL and advanced EGFR-mutant LUAD in elderly have poor outcome. However, the patient was successfully treated with different TKIs without serious adverse events.

## Case presentation

**Fig. 1:**
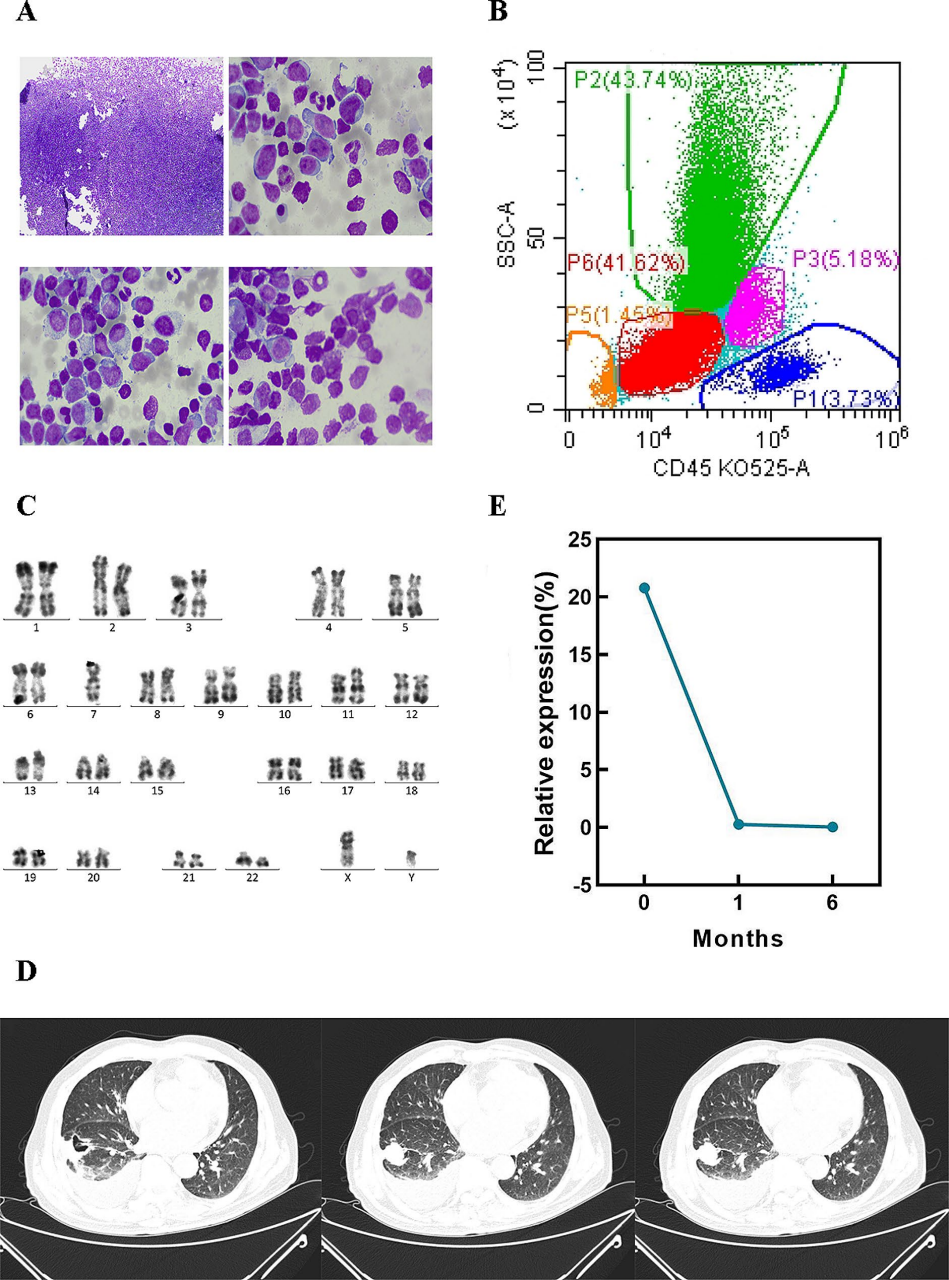
Bone marrow MICM examination and chest CT scan of the patient at initial diagnosis. **(A)** Bone marrow morphology; **(B)** Flow cytometry analysis; **(C)** Chromosome karyotype analysis; **(D)** Chest CT scan (lung window); **(E)** Relative copy of *BCR::ABL1* transcript before and after TKI therapy

An 84-year-old man with fatigue and dizziness for 2 days was admitted to the Affiliated People’s Hospital of Jiangsu University on 17 October, 2022. The blood tests showed white blood cells 105.28 × 10^9^ /L, hemoglobin 119 g/L, platelets 27 × 10^9^ /L. Moreover, 7% of blasts was observed in peripheral blood when conducted a peripheral smear examination. Bone marrow morphology indicated ALL or acute phase of chronic myeloid leukemia accounted for 46.5% of the blasts (Fig. 1A). Flow cytometry analysis showed 41.62% of the lymphoblasts (Fig. 1B) with an immune-phenotype of CD34^+^/CD19^+^/CD10^+^/CD33^+^/CD13^+^/HLA-DR^+^/cCD79a^dim^/ CD11b^−^/cMPO^−^/CD15^−^/CD14^−^/CD38^−^/CD36^−^/CD117^−^/CD123^−^/CD3^−^/cCD3^−^/CD4^−^/CD7^−^/CD20^−^/CD22^−^/CD23^−^/CD16^−^/CD56^−^. The chromosome karyotype was 46, XY, t(9;22)(q34;q11) [4]/45, XY, idem, -7 [6] (Fig. 1C). Real-time quantitative PCR revealed that the relative copy of *BCR::ABL1* (P210) transcript was 20.78% (Fig. 1E). Mutation of TET2 c.4210 C > T (p.Arg1404Ter) with a variant allel frequency (VAF) of 46.3% was also identified by next-generation sequencing (NGS) (222 gene mutation panel). Importantly, the ultrasound examination did not show splenomegaly. Based on the above information, this patient was diagnosed with Ph^+^ ALL. Notably, a chest computed tomography (CT) indicated a space-occupying lesions, characterized by the presence of void, in the right lower lope with the enlargement of mediastinal lymph node and right pleural effusion (Fig. 1D). Tumor markers tests demonstrated that the carcinoembryonic antigen (CEA) was 10.30 ng/mL, whereas the cancer antigen 125 (CA125) was 141.80 U/mL, and the other markers were normal. Regrettably, further biopsy for the pulmonary space-occupying lesion was refused by the patient. The patient was pretreated with cyclophosphamide and prednisone to prevent tumor lysis syndrome when Ph^+^ ALL was diagnosed, and was immediately treated with TKI (flumatinib 600 mg/day) alone. With the continuous treatment, the patient’s symptoms start to show improvement after 10 days, and the blood test was returned to normal after one months. Moreover, the relative copy of *BCR::ABL1* transcript has decreased significantly at the endpoint of 1 month and 6 months (Fig. 1E).

**Fig. 2:**
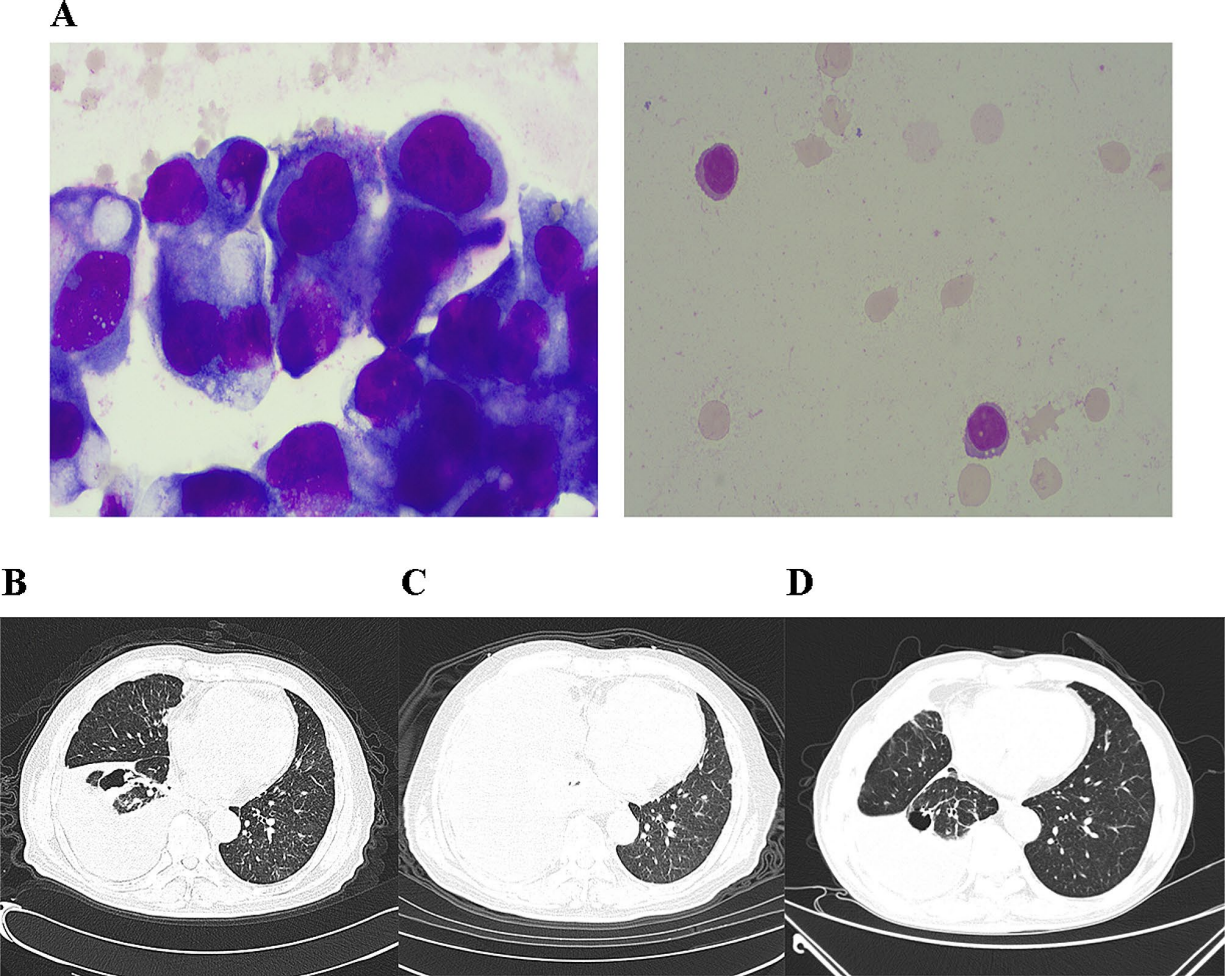
Hydrothorax Cytology examination of and chest CT scan of the patient. **(A)** Cytology examination of hydrothorax; **(B)** Chest CT scan at the diagnosis time of LUAD; **(C)** Chest CT scan at the beginning of EGFR-TKI treatment; **(D)** Chest CT scan after 8 months of EGFR-TKI treatment

On 24 November, 2022, the patient was admitted to our hospital again due to “chest tightness”, and a chest CT indicated that a space-occupying lesions in the right lower lope was roughly similar to the anterior results, but the right pleural effusion with right lower lung atelectasis progressed more than before (Fig. 2B). Thoracentesis was completed, and a routine examination of hydrothorax indicated a bloody, Rivalta test positive, cell count 674.00 × 10^6^ /L, mononuclear cell percentage 90% and multinuclear cell percentage 10%. A biochemical test of hydrothorax demonstrated a total protein 44.10 G/L, adenosine deaminase 5.40 u/L and lactate dehydrogenase 467.00 u/L. A cytology examination observed malignant tumor cells indicated adenocarcinoma (Fig. 2A). The patient was diagnosed with adenocarcinoma of the right lower lope with cancerous hydrothorax. However, the patient still decided to ignore it temporarily. Until 27 January, 2023, the patient experienced severe chest tightness and asthma with chest pain. Repeating chest CT examination indicated the enlarged lesions and increased pleural effusion, which was reconfirmed by cytology examination of the pleural effusion as cancerous hydrothorax caused by LUAD. The patient decided to be treated with pemetrexed (0.8 g) and cisplatin (40 mg) for pleural perfusion. After the treatment, the patient’s symptoms start to show improvement. At the same time, the NGS (58 gene mutation panel) of hydrothorax detected EGFR exon 19 c.2235_2249del p.E746_A750del mutation (VAF 21.52%), that is EGFR exon 19 deletion mutation. On 7 February, 2023, the treatment was adjusted for EGFR-TKI (oxertinib 80 mg/day) and bevacizumab (400 mg). At the endpoint of 8 months after EGFR-TKI treatment, chest CT examination showed a significant narrowing of the lesion (Fig. 2C and D). Until October, 2023, the patient felled good with normal blood test and reduced lung lesions. Ph^+^ ALL reached CR, and LUAD was evaluated as PR. However, the patient was not willing to be admitted to hospital for the evaluation of two diseases.

## Discussion and conclusions

In this report, the case presents with Ph^+^ ALL and EGFR mutant LUAD synchronous overlap, of which pathogenesis is related to abnormal tyrosine kinase activation. As far as we know, this was the first time to report such a case. The potential relationship between Ph^+^ and EGFR mutation in the activation of tyrosine kinase remain poorly revealed. The Ph chromosome is a translocation between chromosome 9 and 22, with the formation of *BCR::ABL1* fusion gene [12]. *BCR::ABL1* fusion gene encodes proteins with unusually high tyrosine kinase activity and activates multiple signaling pathways such as PI3K/AKT, JAK/STAT and their respective downstream targets, leading to the changes in cell proliferation, adhesion and survival properties [12]. EGFR mutation has been identified as one of the most important driver mutations of lung cancer [8, 13]. EGFR belongs to the ERBB family of tyrosine kinase receptors on the cell surface [9, 10]. Upon activation of these receptors with other homodimeric or heterodimeric ERBB members, it triggers the RAS/RAF/MEK and PI3K/AKT/mTOR signaling pathways [9, 10]. It can guide downstream phosphorylation, leading to aberrant tyrosine kinase activation, involved in the proliferation, differentiation, migration, and apoptosis of some cells [9, 10]. Since the two diseases are synchronous overlap, we consider them as independent. However, whether they could affect each other during disease treatment needs further studies. Although Ph^+^ and EGFR mutation cause different diseases in a different manner, they share several common downstream pathways, such as PI3K/AKT signaling. Whether the two different diseases could be solved by one drug needed further functional and clinical studies. Interestingly, a recent study indicated that the crizotinib, a treatment drug approved by the Food and Drug Administration (FDA) for ROS or ALK positive NSCLC, was shown to make therapeutic effects on patients with Ph^+^ leukemia and even *BCR::ABL1*^T315I^ mutation [14]. Moreover, two Phase 1 trial of dasatinib combined with EGFR-TKI in EGFR-mutated lung cancer were conducted, and showed significant anticancer activity [15, 16].

Clinical outcome of both Ph^+^ ALL and advanced EGFR-mutant LUAD in elderly population was extremely poor. It is difficult to give standard doses of chemotherapy for cancer treatment in elderly patients. Inhibition of the activity of abnormal tyrosine kinase caused by *BCR::ABL1* has become an important method for the treatment of Ph^+^ leukemia [17]. EGFR-TKI, a class of compounds that can inhibit the activity of tyrosine kinase enzyme, could be used as a competitive inhibitor of adenosine triphosphate and tyrosine kinase, or as an analogue of tyrosine to block the activity of tyrosine kinase and inhibit cell proliferation [11]. Therefore, EGFR-TKI treatment is also an important therapeutic approach for LUAD with EGFR mutation and provide a significant survival benefit to patients. Based on the above theoretical basis, the patient was successfully treated with different TKIs without serious adverse events (AEs). As is well known, the AEs such as pleural effusion, rash, and cardiac related AE are common in TKIs. However, these common AEs were not occurred in this patient, suggesting a relatively safe for patients using two different TKIs. Accordingly, more cases and longer follow-up time are needed to verify the results.

In summary, as far as we know, it is the first time reported a case of Ph^+^ ALL and EGFR mutant LUAD synchronous overlap, of which pathogenesis is related to abnormal tyrosine kinase activation. This patient was successfully treated with two different TKIs without serious adverse events.

## Data Availability

No datasets were generated or analysed during the current study.

## Abbreviations

ALL: Acute lymphoblastic leukemia
Ph: Philadelphia
TKI: Tyrosine kinase inhibitors
NSCLC: Non-small cell lung cancer
LUAD: Lung adenocarcinoma
EGFR: Epidermal growth factor receptor
MPM: Multiple primary malignant tumors
VAF: Variant Allel Frequency
NGS: Next-generation sequencing
CT: Computed tomography
CEA: Carcinoembryonic antigen
CA125: Cancer antigen 125
FDA: Food Drug Administration
AEs: Adverse events
